# How Stress Affects Your Budget—Stress Impacts on Starch Metabolism

**DOI:** 10.3389/fpls.2022.774060

**Published:** 2022-02-11

**Authors:** Camila Ribeiro, Mark Stitt, Carlos Takeshi Hotta

**Affiliations:** ^1^Centro de Tecnologia Canavieira SA, Piracicaba, Brazil; ^2^Max Planck Institute for Molecular Plant Physiology, Potsdam, Germany; ^3^Departamento de Bioquímica, Instituto de Química, Universidade de São Paulo, São Paulo, Brazil

**Keywords:** abiotic stress, biotic stress, starch, circadian clock, starch metabolism

## Abstract

Starch is a polysaccharide that is stored to be used in different timescales. Transitory starch is used during nighttime when photosynthesis is unavailable. Long-term starch is stored to support vegetative or reproductive growth, reproduction, or stress responses. Starch is not just a reserve of energy for most plants but also has many other roles, such as promoting rapid stomatal opening, making osmoprotectants, cryoprotectants, scavengers of free radicals and signals, and reverting embolised vessels. Biotic and abiotic stress vary according to their nature, strength, duration, developmental stage of the plant, time of the day, and how gradually they develop. The impact of stress on starch metabolism depends on many factors: how the stress impacts the rate of photosynthesis, the affected organs, how the stress impacts carbon allocation, and the energy requirements involved in response to stress. Under abiotic stresses, starch degradation is usually activated, but starch accumulation may also be observed when growth is inhibited more than photosynthesis. Under biotic stresses, starch is usually accumulated, but the molecular mechanisms involved are largely unknown. In this mini-review, we explore what has been learned about starch metabolism and plant stress responses and discuss the current obstacles to fully understanding their interactions.

## Introduction

**FIGURE 1:**
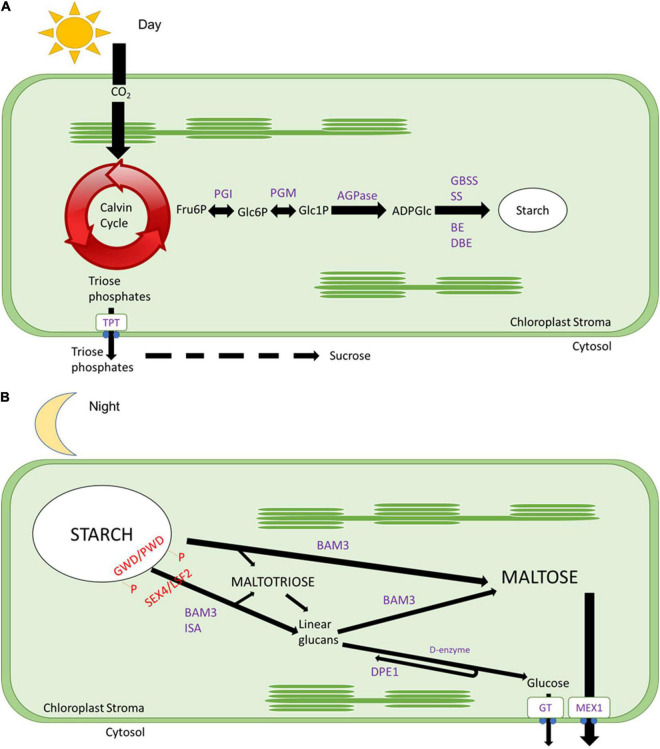
Daily starch metabolism. **(A)** During the day, CO_2_ is fixed by the Calvin cycle, and trioses phosphates will be exported out of the chloroplast to the cytosol through a triose phosphate transporter protein to be converted into sucrose. While fructose 6-phosphate will be converted into glucose 6-phosphate by phosphoglucose isomerase (PGI), then converted to glucose 1-phosphate by phosphoglucomutase (PGM), later converted to ADP glucose by ADP glucose pyrophosphorylase (AGPase), after polymerised by starch synthases (SS) and granule bound starch synthases (GBSS) and branched by branching enzymes (BE) and debranching enzymes (DBE). **(B)** During the night, the surface of the starch granule is loosed by glucan phosphorylation catalysed by glucan water dikinases (GWD), and phosphoglucan water dikinases (PDW) followed by the action of β-amylases (BAMs, especially BAM3 with a subsidiary role for BAM1, see Smith and Zeeman, 2020) and isoamylase 3 (ISA3). Starch breakdown results in the formation of maltose and maltotriose. Maltotriose is converted by disproportionation enzyme 1 (DPE1) to glucose and longer glucans that can be degraded to maltose by β-amylases. The action of BAM3, ISA3, and PDE1 requires removal of phosphate by the glucan phosphatases starch excess 4 (SEX4) and Like Sex Four 2 (LSF2). Glucose is exported to the cytosol by glucose transporter (GT), and maltose is exported out by a maltose exporter 1 (MEX) to be utilised as an energy source for nighttime reactions.

**FIGURE 2:**
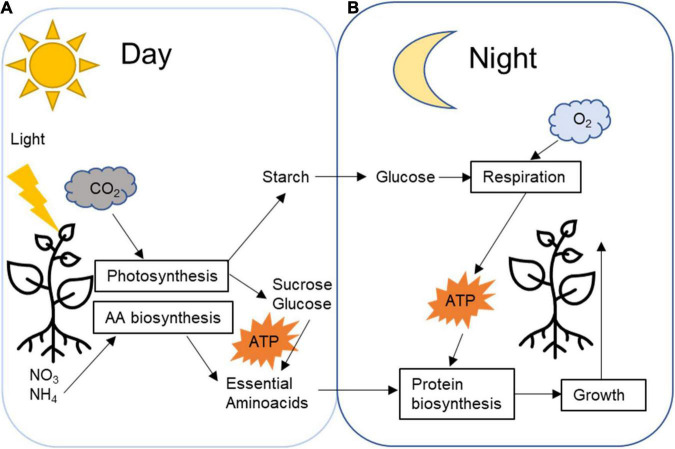
Plant daily transitory starch carbon management processes. **(A)** During the day, CO_2_ is fixed by the Calvin cycle generating sugar that will be promptly utilised as an energy source for day metabolism, such as nitrite and nitrate fixation and amino acids biosynthesis. Part of the fixed carbon is temporarily stored in the plastids as starch. **(B)** During the night, starch is broken down into glucose that respiration will generate energy for nighttime metabolism reactions, such as protein biosynthesis and plant growth.

Energy management is vital for plant development, and it is diversely regulated across species depending on life forms and environmental conditions. Photosynthetic reactions in leaves generate carbohydrates that can be immediately utilised as an energy source. However, part of the photosynthetic products in most plants will be stored as transitory starch during the daytime (Figure 1A; Stitt and Zeeman, 2012; Smith and Zeeman, 2020). During the nighttime, the starch is broken down (Figure 1B) to provide a source of carbon for continued sucrose synthesis and export and respiration, thus fueling the synthesis of protein and other cellular components, growth and development throughout the whole 24-h cycle (Figure 2; O’Leary et al., 2017; Smith and Zeeman, 2020). The rate of degradation during the nighttime is regulated so that starch is almost depleted at dawn when photosynthesis resumes and a new cycle begins (Smith and Stitt, 2007; Graf et al., 2010). The circadian rhythm highly regulates this carbon management process.

More generally, starch acts as a sugar source when photosynthesis is impaired or unavailable, not only in the nighttime but also during seed germination, tuber sprouting, tissue regeneration, or under stress conditions (MacNeill et al., 2017; Smith and Zeeman, 2020). Starch can also have specialised roles: e.g., in the guard cells, starch can be degraded during the daytime to promote rapid stomatal opening (Valerio et al., 2011; Flütsch et al., 2020). Accordingly, starch can be stored to be used as a reserve in different timescales. Usually, transitory starch is synthesised and degraded within a day. In contrast, long-term starch is stored, often outside the source organ, to support vegetative or reproductive growth, reproduction, or stress responses (MacNeill et al., 2017).

Stress can affect carbon metabolism by affecting photosynthetic rate, carbon allocation, and night respiration. These impacts can reduce plant growth and development depending on the type of stress and affected tissue. Studies related to the effects of stress on starch metabolism have faced significant challenges because the response depends on the nature, strength and duration of the stress, how gradually it develops, and plant developmental stage and the time of the day and (Köhl, 2016). In addition, experiments on stress responses are usually not standardised, making it difficult to compare different studies. Furthermore, as transitory starch is in constant flux, experiments that measure starch at only one or a few time points may not capture complex responses on the diel rhythms. Finally, it is now clear that other degradation pathways can operate under stress conditions in the light in addition to the daily nocturnal degradation pathway.

## Starch Metabolism and Abiotic Stress

Changes in the plant starch metabolism due to abiotic stress depend on how the stress affects growth, the relative extent of the inhibition of growth and photosynthesis, and whether modifications in C allocation support stress responses. Each of these factors depends on the type of the stress, its intensity and duration. Early stress responses require resources to provide energy and support the synthesis of new molecules to protect, restore, and acclimate the plant. As photosynthesis is frequently impaired by stress, an important role is played by carbon reallocated from starch, avoiding a significant reorganisation of metabolism (Hummel et al., 2010). Starch synthesis is decreased in water and temperature stress, mainly due to stomatal closing and lower rates of photosynthesis (Zrenner and Stitt, 1991; Thitisaksakul et al., 2012). However, there are also situations in which the stress arrests growth without affecting photosynthesis, leading to an overall increase in starch reserves (Hummel et al., 2010; de Morais et al., 2019).

As recently reviewed, transitory starch content is usually observed to decline in leaves in response to salt, drought, and cold stress (Thalmann and Santelia, 2017; Dong and Beckles, 2019), consistent with the idea that starch is synthesised at lower rates and/or is broken down more rapidly to redirect carbon for stress responses. An example of the increased degradation under stress is the stimulation of starch breakdown even under mild drought (Zrenner and Stitt, 1991) and by low temperature (Kaplan and Guy, 2004). Together, this allows carbon to be reallocated to make osmoprotectants or cryoprotectants that promote osmotic adjustment and stabilise proteins (Kempa et al., 2008; Krasensky and Jonak, 2012; Tarkowski and Van den Ende, 2015; Zanella et al., 2016); scavengers of free radicals (Couée et al., 2006; Keunen et al., 2013); and signals that refine stress responses (Rolland et al., 2006; Rook et al., 2006).

Starch degradation in response to stress may use different combinations of enzymes, while nighttime degradation uses mainly BAM3 and ISA3. Under water, stress amylase 3 (AMY3) and BAM1 are induced (Thalmann and Santelia, 2017). BAM1 can be upregulated by temperature, osmotic and salinity stress in leaf guard cells and roots (Kaplan and Guy, 2004, 2005; Kempa et al., 2008; Valerio et al., 2011). BAM1 protein is regulated by reduced thioredoxins, which are light-dependent, possibly counteracting starch synthesis during the daytime (Valerio et al., 2011; Zanella et al., 2016). BAM1 and α-amylase 3 (AMY3) promote daytime starch degradation to support proline biosynthesis in mesophyll cells under osmotic stress (Zanella et al., 2016). In guard-cells, BAM1-dependent starch degradation promotes stomatal opening in diel rhythms in response to osmotic stress (Valerio et al., 2011).

In some experiments, plants accumulated starch in response to stress (Thalmann and Santelia, 2017). This apparent contradiction is associated with the level of stress and timing of the measurements. For example, in early stress, starch degradation may predominate as C is mobilised to support for adaptive responses; in mild drought and salt stress-responses, starch may accumulate because growth is inhibited, but photosynthesis not is proportionately affected (Hummel et al., 2010; de Morais et al., 2019). While in severe stress, such as high temperatures associated with drought during grain filling, starch degradation may predominate because carbon assimilation is heavily affected due to stomata closure or damage to the photosystems (Bahuguna et al., 2017; Dong and Beckles, 2019). As an alternative, starch accumulation under salt stress has been suggested to play a role in capturing Na^+^ in its granules (Kanai et al., 2005).

The hormone abscisic acid (ABA) promotes stomatal closure under water, temperature, and osmotic stresses, lowering the internal leaf CO_2_ and inhibiting photosynthesis. However, in these conditions, starch degradation in the light allows maintenance of Calvin-Benson Cycle metabolite levels and, hence, rapid flux in the Calvin-Benson cycle to generate RuBP that supports rapid oxygenation of RuBP and photorespiration (Weise et al., 2006; Sharkey, 2019; Stitt et al., 2021). Photorespiration can aid energy dissipation under stress by regenerating ADP and NADP, avoiding ROS formation and overreduction of the chloroplastidal electron transport chain, which results in photoinhibition (Kozaki and Takeba, 1996; Timm et al., 2019; Timm and Hagemann, 2020).

Starch can also be stored outside source organs as a reserve to be used in a situation of longer-term low carbon assimilation. Reproductive organs seeds or tubers can accumulate large amounts of starch to support the growth of the next generation. However, starch is often accumulated outside source leaves during vegetative growth and can play an essential role in stress responses. A noteworthy example is starch reserves in the woody tissues of the trees, in the xylem-ray parenchyma cells (Noronha et al., 2018). While little is known about the genes involved in the synthesis and degradation of starch in these tissues, starch reserves are pivotal for cold tolerance in the winter and budding in the spring (Sauter, 1988; Witt and Sauter, 1994; Noronha et al., 2018). Embolised conduits can be refilled at nighttime, but this requires much energy and solutes, provided from starch degradation, especially when the soil is dry and photosynthesis is inhibited (Zwieniecki and Holbrook, 2009). As drought stress can lead to hydraulic failure due to cavitation and conduit embolism, starch has a significant role in preventing tree deaths (McDowell et al., 2011).

Long term starch can also be stored in source organs. In *Zea mays* L. (maize, Poaceae), unlike the starch in mature zones of the leaves, starch levels in the growth zones are kept high at nighttime (Czedik-Eysenberg et al., 2016). This starch can be used to support leaf growth in the first hours when nighttime is extended, showing that this is a mechanism to buffer against stresses that limit carbon assimilation (Czedik-Eysenberg et al., 2016).

During the late reproductive phase, plants under stress may use vegetative starch reserves to guarantee the complete development of their seeds (Trouverie et al., 2006; Cuellar-Ortiz et al., 2008). In grain crops, reallocation of carbon in response to abiotic stress can also lead to grain abortion and a decrease in grain starch (Andersen et al., 2002; Mangelsen et al., 2011). The regulation of starch synthesis in seeds may differ from that in leaves. For example, AGPase stability is drastically reduced by high temperature in maize and *Hordeum vulgare* L. (barley, Poaceae), reducing grain starch (Singletary et al., 1994; Wallwork et al., 1998; Linebarger et al., 2005). In *Triticum aestivum* L. (wheat, Poaceae) and *Oryza sativa* L. (rice, Poaceae), high temperatures reduced the transcript levels of several starch synthesis genes, which are correlated with a reduction in seed size (Hurkman et al., 2003; Yamakawa and Hakata, 2010). Thus, changes in starch metabolism due to abiotic stress can also affect the quality and productivity of crops.

## Starch Metabolism and Biotic Stress

Biotic stress can also impact starch metabolism. In contrast to most abiotic stresses, starch is accumulated, often characterised as a symptom of pathogen infection. Abnormal starch accumulation has been described in different types of plant-pathogen responses, such as *Puccinia hordei* Otth. (brown rust, Basidiomycota) infecting barley (Scholes and Farrar, 1987), *Plasmopara viticola* (Berk. and M.A. Curtis) Berl and De Toni (downy mildew, Oomycota) infecting *Vitis vinifera* L. (grapevine, Vitaceae) (Gamm et al., 2011), *Plasmodiophora brassicae* Woronin (clubroot disease, Cercozoa) infecting Brassicaceae (Ludwig-Müller and Schuller, 2008), as well as tobacco mosaic virus (mottled browning) in *Nicotiana tabaccum* L. (tobacco, Solanaceae) (Allan et al., 2001; Zhao et al., 2016).

Starch accumulation due to biotic stress has been explored in detail in *Citrus* spp. L. (Rutaceae) infected with *Candidatus* Liberibacter, which causes citrus greening or Huanglongbing (HLB), due to the economic impact of this disease in worldwide orange production (Etxeberria et al., 2009; Fan et al., 2010; Gonzalez et al., 2011). HLB is known to cause abnormal callose accumulation in citrus phloem tissues impairing source to sink flux, leading to decreased fruit production and eventually tree decay (Koh et al., 2012; Wang et al., 2017; Achor et al., 2020). Different starch biosynthetic genes were upregulated in response to HLB in leaves, such as starch synthases, granule bound starch synthase, and ADP-glucose pyrophosphorylase (Albrecht and Bowman, 2008; Martinelli et al., 2012; Mafra et al., 2013). Excessive starch accumulation in the chloroplasts is hypothesised to damage them and restrict CO_2_ diffusion (Lemoine et al., 2013). It has also been proposed that excessive starch accumulation is due to the stimulated entry of carbon from the cytosol via a plastid envelope glucose-6-phosphate transporter protein (Martinelli et al., 2012). The putative crucial role of this transport protein is supported by the lack of regulation of this gene in symptomatic fruits, where starch is not accumulated (Martinelli et al., 2012, 2013; Martinelli and Dandekar, 2017).

Curiously, HLB is associated with the induction of starch biosynthesis proteins and with the induction of α-amylase, β-amylase, and phosphoglucan dikinase in leaves (Albrecht and Bowman, 2008; Martinelli et al., 2013; Balan et al., 2018). In healthy plants, these enzymes are commonly expressed at nighttime (Graf and Smith, 2011; Lloyd and Kötting, 2016) but can be expressed more strongly to generate soluble sugars in response to stress (Doyle et al., 2007). In contrast, when quantifying sugars, an increase in maltose was identified in symptomatic leaves, with a decreased expression of MEX1 (Fan et al., 2010). Therefore, it is difficult to formulate a clear account of how HLB affects the daily rhythms of leaf starch without a full time course.

Transient expression of the truncated effector of LasΔ5315 bacteria in *Nicotiana benthamiana* Domin (Solanaceae) resulted in excessive starch accumulation and overexpression of genes related to the starch synthesis (Pitino et al., 2018). Likewise, fungal volatiles emitted by *Alternaria alternata* (Fr.) Keissl. (leaf spot, Ascomycota) induce abnormal starch accumulation in *Arabidopsis thaliana* (L.) Heynh. (Brassicaceae) and *Solanum tuberosum* L. (potato, Solanaceae) (Ezquer et al., 2010; Li et al., 2011), that is linked with induction of potato SS classes III and IV, and plastidial changes in redox status of plastidial enzymes mediated by NADP-thioredoxin reductase (Ezquer et al., 2010; Li et al., 2011).

## Circadian Rhythms and Stress Responses

Starch levels and gene expression are often analysed only once a day, and the actual time of day is rarely specified, with few exceptions (Quick et al., 1992; Thalmann et al., 2016). However, starch metabolism and plant stress responses typically underly rhythms with a period close to 24 h. Many related biological processes, such as photosynthesis, resistance to abiotic and biotic stresses, floral induction by photoperiodism, petal movement and floral fragrance exhibit circadian rhythms. Further, it is known that disorders in circadian function reduce plant growth and function (Dodd et al., 2005; McClung, 2019).

The circadian clock synchronises endogenous events with environmental rhythms, including responses to stress. For example, in gating, the same environmental signal may lead to different responses at different times of the day (Hotta et al., 2007; Seo and Mas, 2015). A study in Arabidopsis found 33 genes differentially expressed in dry conditions at midday, but 508 genes differentially expressed at the end of the light period, just 6 h later (Wilkins et al., 2010). In turn, responses to stress can also regulate the circadian oscillator. ABA may be part of a small regulatory loop, as the central oscillator component LATE ELONGATED HYPOCOTYL (LHY) regulates ABA biosynthesis (Adams et al., 2018), while ABA upregulates another central oscillator component, TIMING OF CAB EXPRESSION 1 (TOC1), in a clock-controlled manner (Legnaioli et al., 2009). The expression of the central oscillator *COMPONENT CIRCADIAN CLOCK ASSOCIATED 1* (*CCA1*) is affected by induction with the flg22 peptide and *P. syringae* infection, while *LHY* and *TOC1* show salicylic acid-induced increased gene expression (Lai et al., 2012; Zhang et al., 2013).

The circadian clock also regulates starch metabolism (Lu et al., 2005; Mugford et al., 2014; Seki et al., 2017; Flis et al., 2019). Such regulation avoids starvation stress at the end of the nighttime while providing abundant sucrose for maintenance and growth across different photoperiods (Flis et al., 2019). The amount of starch accumulated and its rate of mobilisation are regulated such that starch is consumed at around dawn, which the circadian clock can anticipate (Graf and Smith, 2011; Scialdone et al., 2013). Mutants of different circadian clock components fail to distribute starch mobilisations correctly, either consuming reserves too quickly, leading to late-night-time carbon deficits and transient starvation, or too slowly, leading to accumulation of starch (Eimert et al., 1995; Messerli et al., 2007; Graf and Smith, 2011; Scialdone et al., 2013; Flis et al., 2019). Thus, any circadian clock changes caused by stresses may affect the dynamics of starch rhythms.

Transcriptomic studies of Arabidopsis and sugarcane leaves showed that the transcription of genes associated with starch degradation enzymes peak at dusk and decrease at dawn (Harmer et al., 2000; Smith et al., 2004; Usadel et al., 2008; Hotta et al., 2013). However, gene expression may not correlate with protein abundance or enzyme activity. In particular, whilst many transcripts show marked oscillations, the abundance of their encoded proteins are often relatively stable across the 24 h cycle, raising questions about the biological function of these oscillations in transcript abundance (Baerenfaller et al., 2012; Ponnala et al., 2014; Graf et al., 2017). In addition to regulating expression, the circadian clock also buffers the starch metabolism against sudden fluctuations in light and temperature (Graf et al., 2010; Pyl et al., 2012; Pilkington et al., 2015; Flis et al., 2019).

There are two models to explain how rhythms in starch metabolism are generated: one that integrates starch abundance and timing information (Scialdone et al., 2013; Pokhilko et al., 2014) and one that proposes continuous regulation of the circadian clock by signals from sucrose or related metabolites (Webb and Satake, 2015; Seki et al., 2017). In addition, it has been proposed that increased rates of starch mobilisation with time in the light result in an endogenous glucose-6-phosphate oscillation (Flis et al., 2019) that serves as a buffer to regulate carbon reserves from photosynthesis at dusk. Even so, the molecular mechanisms of this control are poorly understood.

Low-carbon availability regulates the REVEILLE family, regulating many circadian clock genes (Moraes et al., 2019). Carbon starvation can also regulate the circadian clock, triggered by growth under low light or low CO_2_ (Haydon et al., 2013; Frank et al., 2018). In these conditions, basic leucine zipper 63 (bZIP63) upregulates the circadian oscillator gene pseudo-response regulator 7 (*PRR7*) (Frank et al., 2018). As the circadian clock and SnRK1 regulate bZIP63, it may connect the circadian clock and sugar signalling (Mair et al., 2015; Viana et al., 2021). Mutants of *bZIP63* exhibit impaired growth under light/dark cycles but not under constant light, possibly because starch degradation is accelerated, leading to starvation stress by the nighttime’s end (Viana et al., 2021). Interestingly, *bZIP63* is also regulated by ABA (Matiolli et al., 2011). Considering that bZIP63 forms heterodimers with other family members, like bZIP1 (Kang et al., 2010), and OsZIP23 may play a similar role in rice (Kim et al., 2017), the bZIP family of transcription factors may be at the centre of three major regulatory networks.

## Future Prospects

Starch can be an essential carbon source when photosynthesis is inhibited at night and under many stress conditions. The carbon derived from starch may help support some continued growth, but it is probably even more critical because it supports metabolic and cellular responses that ameliorate stress. While the pathways of starch degradation are pretty well-understood in source leaves, less is known about the enzymes involved in starch metabolism in sink tissues under abiotic stress. Furthermore, little is known about the interaction between starch turnover and stress responses, especially when another regulatory pathway, the circadian clock, is involved. In biotic stresses, the molecular mechanisms involved in starch accumulation are mainly unknown. In general, a better understanding of the dynamics of regulators of starch metabolism under different types of stress and at different stress intensities is needed, especially post-transcriptional regulators. A better understanding of how starch is used during different stresses could allow breeding programs or genetic engineering to generate stress-resilient plants, especially starch-based feedstocks.

## Author Contributions

CR, MS, and CH wrote and edited the manuscript. All authors contributed to the article and approved the submitted version.

## Conflict of Interest

CR was employed by Centro de Tecnologia Canavieira SA. The remaining authors declare that the research was conducted in the absence of any commercial or financial relationships that could be construed as a potential conflict of interest.

## Publisher’s Note

All claims expressed in this article are solely those of the authors and do not necessarily represent those of their affiliated organizations, or those of the publisher, the editors and the reviewers. Any product that may be evaluated in this article, or claim that may be made by its manufacturer, is not guaranteed or endorsed by the publisher.
